# *GSTM1* polymorphism contribute to colorectal cancer in Asian populations: a prospective meta-analysis

**DOI:** 10.1038/srep12514

**Published:** 2015-07-29

**Authors:** Jing Li, Wen Xu, Fang Liu, Silin Huang, Meirong He

**Affiliations:** 1 Guangdong Provincial Key Laboratory of Gastroenterology, Department of Gastroenterology, Nanfang Hospital, Southern Medical University, Guangzhou 510515, Guangdong Province, China

## Abstract

Glutathione *S*-transferases (GSTs) are enzymes which expressed in many tissues and play important roles in neutralization of toxic compounds, and protecting hosts against cancer. Among several GSTs, Glutathione *S*-transferases mu (GSTM) has been drawn attention upon the association with the genetic risk for many types of cancers. But whether the *GSTM1* polymorphisms confer the susceptibility to colorectal cancer in Asians has not been well established. We searched the PubMed database with *GSTM1*, polymorphism and colorectal cancer, attempting to identify the eligible studies. In total, 33 case-control studies in Asian populations with 8502 colorectal cancer patients and 13699 controls were included in the current meta-analysis. The association between the polymorphism and susceptibility to colorectal cancer was evaluated by the odds ratio (OR) and 95% confidence intervals (CI). The pooled meta-analysis suggested that *GSTM1* null variant was correlated to the colorectal cancer risk in Asians. There was a marginal heterogeneity among these eligible studies. Nevertheless, cumulative meta-analysis observed a trend of an obvious association between the *GSTM1* null genotype and colorectal cancer risk in Asians. In summary, the meta-analysis suggested that *GSTM1* null polymorphism confer the susceptibility to colorectal cancer in Asians, especially in Chinese populations.

Colorectal cancer is the second leading common cancer and a major cause of cancer-related deaths in the world-wide, which accounts for about 9.7% death rate of all cancers^1^,^2^. It has been reported that about 1.2 million new cases of colorectal cancer in the world in 2008^2^. Although the incidence rate of colorectal cancer decreases in the western developed countries^3^,^4^, the rate still rises in the developing countries, especially in China. The etiology and development of colorectal cancer was not fully understood yet, but considering the previous studies, the colorectal cancer was proven to be complicated and multifactorial cancer^5^,^6^. Previous evidence suggested that the environmental risk factors and genetic factors both affected the pathogenesis of colorectal cancer^7^. It was reported that familial colorectal cancer accounts for around 5–15%^8^. Fearon ER and his colleagues^6^ found that many mutants/variants contribute to the pathogenesis of the sporadic and inherited forms colorectal cancer, including several GSTs variants.

GSTs are a super-family of phase II detoxification enzymes which play critical roles in the detoxification of exogenous and endogenous reactive species through conversion of toxic compounds to hydrophilic metabolites^9^,^10^. To date, there are 8 classes of GSTs including alpha (α), kappa (κ), mu (μ), omega (ω), pi (π), sigma (σ), theta (θ) and zeta (ζ) have been clarified^11^, and polymorphisms have been identified among several of them^12^. Glutathione *S*-transferase M1 (*GSTM1*) encodes the mu class of GSTs, which plays vital roles in protecting hosts against cancers^13^. The mu GST enzymes were demonstrated to be more effective at the process of detoxifying cytotoxic and genotoxic reactive species than other GSTs^13^. The null variant (GSTM1*0 allele) is the most common variant of the *GSTM1*, leading to the loss of enzyme activity, and the variant-carriers were proven to be associated with increased risk to cancers^9^. *GSTM1* has been identified to be involved in the pathogenesis and development of certain cancers, inclusive of colorectal cancer^14^.

Many association studies and evidence were conducted and highlighted the *GSTM1* null variant was correlated with the risk of cancers^15^,^16^. Moreover, the *GSTM1* null genotype has been demonstrated to be linked to early onset for colorectal cancer^17^. However, the previous studies which aimed to investigate the association of *GSTM1* polymorphism and the colorectal cancer susceptibility in Chinese populations were controversial, and so were the genetic results in Asians. Inclusive of 33 case-control studies, the present meta-analysis was performed to further explore the relationship between *GSTM1* null variant and the susceptibility to colorectal cancer more comprehensively in Asians.

## Methods

### Literature search and inclusion criteria

We systematically searched the PubMed database (http://www.ncbi.nlm.nih.gov/pubmed/) to identify the eligible case-control studies using the following keywords: (“glutathione *S*-transferase” or “GST” or “glutathione *S*-transferase M1” or “*GSTM1*”), (“polymorphism” or “Single Nucleotide Polymorphism”) and (“colorectal cancer” or “CRC” or “colorectal carcinoma” or “colorectal adenoma”). There was no language limitation in the literature search. Titles and abstracts of these searching studies were primarily screened and full papers were further retrieved to confirm eligibility, the reference lists were also examined to find other relevant studies. These studies were reported from 1996 to 2014. These given studies included into our meta-analysis had to meet the following inclusion criteria: (1) case-control validation study, (2) the studies should estimate the association of the *GSTM1* null variant with risk to colorectal cancer, (3) the studies should provide odds ratio (OR) with 95% CI or available data, (4) the studies should be conducted in Asian populations. Reviews and duplicate studies were excluded from the analysis. If the same case-control study were overlapped in multiple publications, only the most complete or most recent literature was included in the present study.

### Data extraction

The available data originated in the eligible studies was independently extracted by two co-authors. The following information was collected: first author’s name, year of publication, country of the study conducted, ethnicity of participants, numbers of cases and controls and the genotype distributions of *GSTM1* null variant. All subjects followed the principles of the Declaration of Helsinki.

### Statistical analysis

The association between *GSTM1* null genotype with colorectal cancer risk in Asians was calculated by pooled OR with 95% CI. The ORs were evaluated according to the extracted data. A 95% CI was used for standard for statistical significance and 95% CI without 1 for OR indicated that the genotype may increase or decrease the cancer genetic risk significantly. Firstly, the *I*^*2*^ statistic was estimated to quantify the heterogeneity among all eligible studies^18^, and an *I*^*2*^ < 50% suggested low heterogeneity. In general, the meta-analysis was performed using fixed-effect^19^ or random-effect^20^ models according to the effect estimates in the presence (*I*^*2*^ ≤ 50%) or absence (*I*^*2*^ > 50%) of significant heterogeneity. So, the random-effect model should be chosen once the obvious heterogeneity was observed. Interesting, the heterogeneity was also considered to be significant when *P* < 0.10^18^,^21^,^22^. But it was widely accepted that the cutoff for significance of heterogeneity is *P* < 0.05 in the meta-analysis^23^. Furthermore, Sensitivity analysis was conducted by omitting those studies in turns to estimate the overall pooled ORs in the present study. Additionally, the Begg’s funnel plot and the Egger’s regression plot were considered to be preferred method to assess the publication bias^24^,^25^. An asymmetric funnel plot suggested a relationship between effect and study size, indicating the possibility of either publication bias or a systematic difference between smaller and larger studies^26^,^27^. We further performed a cumulative meta-analysis to investigate a framework for updating a case-control-effect from all eligible studies and to assess how much the genetic effect changes as statistical power accumulates, and to find the trend in risk effect^28^. In the cumulative meta-analysis, studies were ordered by publication year, and the pooled ORs were calculated at the end of each study. All statistical analyses were assessed by using STATA software, version 12.0 (StataCorp LP, College Station, TX, USA). All *P* values were two-sided.

## Results

### Characteristics of the case-control studies

**Figure 1:**
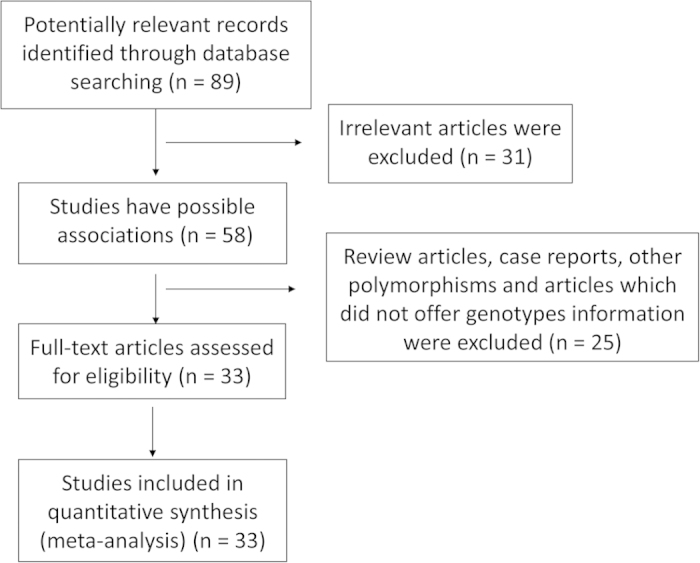
Flow chart of eligible studies.

The flow chart of eligible studies was shown in Fig. 1. Firstly, 89 potential studies were screened after literature search followed with the search strategy, but only 33 eligible studies remained according to the inclusion criteria. All these association studies were published from 1996 to 2014, and 19 studies were in English, and the other 14 studies were published in Chinese, which shown in the Table 1. In total, there are 22201 subjects, comprising of 8502 colorectal cancer patients and 13699 matched controls were included in the current study from these 33 studies. The characteristics of those eligible studies in Asians were shown in Table 1.

### Overall analysis

**Figure 2:**
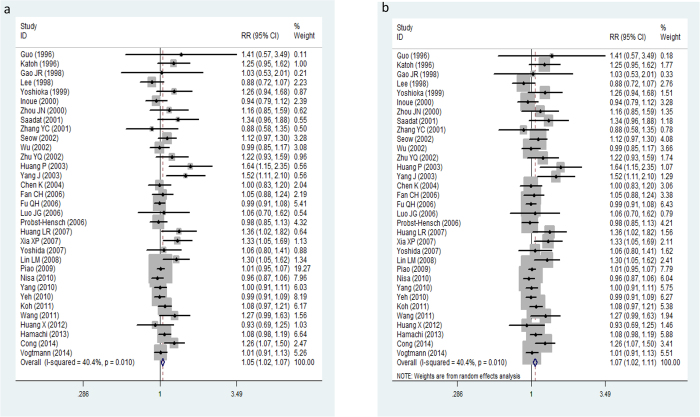
Forest plot for the association between *GSTM1* null polymorphism and colorectal cancer in Asians under fixed-effect model (**a**) and random-effect model (**b**).

There was a marginal heterogeneity among these 33 validation studies in Asian cohorts (*P* = 0. 01, I^2^ = 40.4%) (Fig. 2). Overall, the pooled meta-analysis of eligible case-control studies suggested that *GSTM1* null genotype was significantly associated with the risk to colorectal cancer in Asian populations (Z = 3.32, *P* = 0.001, OR = 1.05, 95% CI: 1.02–1.07) in a fixed-effect model (Fig. 2a). The association still remained (Z = 3.17, *P* = 0.002, OR = 1.07, 95% CI: 1.02–1.11) (Fig. 2b) under the random-effect model. Sensitivity analyses by omitting one study at a time did not materially alter the overall pooled ORs (supplementary Figure S1).

**Figure 3:**
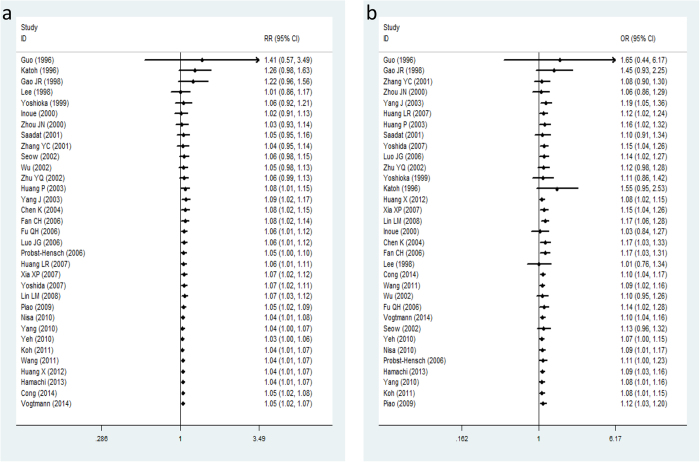
Forest plot in the cumulative meta-analysis accumulated by publication year (**a**) and sample size (**b**).

The cumulative meta-analysis further showed a trend of an obvious association between the *GSTM1* null genotype and colorectal cancer risk in Asians as information accumulated by year (Fig. 3a), moreover, the cumulative analysis accumulated by the sample size also supported the result (Fig. 3b).

### Publication bias

**Figure 4:**
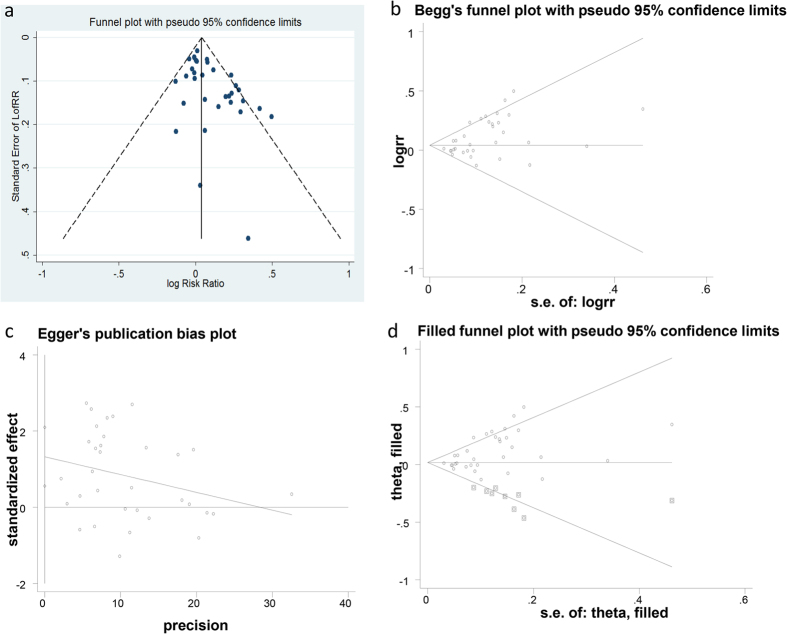
Publication bias test for the meta-analysis: Funnel plot (**a**), Begg’s funnel plot (**b**), Egger’s test (**c**) and Trim and fill test (**d**).

The funnel plot, Begg’s adjust rank correlation test, Egger’s regression test, trim and fill method are four corresponding methods applied to assess the publication bias in the current meta-analysis. Funnel plot, Begg’s funnel plot, Egger’s regression, plot trim and fill funnel plot were produced by these four methods respectively (Fig. 4a–d). There was no obvious asymmetry the shape of the funnel plot and the Begg’s funnel plot (Fig. 4a). However, publication bias was marginal when the Begg’s rank correlation method (z = 2.34, *P* = 0.019 < 0.05) was used, the publication bias was more significant in the Egger’s regression test (t = 3.51, *P* = 0.001 < 0.05) (supplementary Figure S3). Nine missing studies should be filled in the trim and fill method, furthermore, LogRR and its 95%CI altered significantly after the application of trim and fill method (supplementary Figure S4). The combined analyses indicated that there is publication bias in the present study.

### Sub-group analysis

There were 21 studies of Chinese, 6 in Japanese, 2 of Taiwan subjects, each single study in Korean, Singapore, India and Iran in the meta-analysis. Sub-analysis based on the different countries was conducted to verify the heterogeneity, it seemed that there may exist a marginal heterogeneity in Chinese populations (*P* = 0. 023, I^2^ = 42%)

## Discussion

The etiology of colorectal cancer has not been well established, and previous evidence suggested that the pathogenesis of colorectal cancer was intricate and influenced by complicated interactions between genetic factors and environmental risk. Previous studies reported that the *GSTM1* null genotype was in high prevalence in human population, about 40–60% in Europeans^29^ and about 50% in Asians^30^. It has been widely accepted that some susceptible genes correlated to colorectal cancer risk, besides smoking, diet and other environmental factors. Up to date, xenobiotic-metabolizing enzymes were primarily concerned on the pathogenesis of cancers^31^.

GSTM1 is one of the most main subtypes GSTs, which are more effective on protecting host from cancer than others^13^. *GSTM1* null genotype is the most common polymorphism and has been proven to be associated with risk of several cancers^15^,^16^, including colorectal cancer^17^. There were many association studies on relationship between *GSTM1* null polymorphism and colorectal cancer in various ethnicities^32^. Previous validation evidence and meta-analysis studies supported that the *GSTM1* null genotype was significantly related to increased risk to colorectal cancer in Caucasian populations^32^, but the association in Asians has not been revealed yet. Large amount of validation studies have been conducted to detect the association in Asians, but produced inconsistent results. Therefore, we preformed the current meta-analysis to further explore whether the *GSTM1* null polymorphism associated with the susceptibility to colorectal cancer.

In our meta-analysis, 33 eligible studies with 8502 colorectal cancer patients and 13699 healthy controls were pooled to calculate the association between *GSTM1* polymorphism and colorectal cancer. Significant association between *GSTM1* null variant and colorectal cancer in Asian populations was observed in the pooled meta-analysis under both fixed-effect model and random-effect model (Fig. 2a,b). Even though publication bias analyses suggested that obvious bias may exist in the included studies (Fig. 4a–d). The sensitivity analysis indicated the results of meta-analysis were credible and stable (supplementary Figure S1). Furthermore, the cumulative meta-analysis accumulated by publication year or the sample size further confirmed the obvious association of the *GSTM1* null variant colorectal cancer in Asians (Fig. 3a,b). In summary, the meta-analysis verified that the *GSTM1* null variant was linked to the genetic risk of colorectal cancer in Asians, which in accordance with the previous meta-analysis study^26^.

Compared to the previous meta-analyses, more validation studies were pooled in the present meta-analysis. The pooled meta-analysis, the cumulative meta-analyses by publication year and the sample size, and the sensitivity analysis correspondingly supported the *GSTM1* null polymorphism contribute the genetic risk to colorectal cancer in Asians, especially in Chinese. Nevertheless, it should be noted that there are several limitations in the meta-analysis. First, the association between *GSTM1* null variant and colorectal cancer was the only aspect we focused on in this meta-analysis, but the other potential susceptible factors, such as age, sex and smoking status were not considered in the current study, because great majority of these eligible studies did not provide the available information or data. Second, there exist a marginal heterogeneity in Asians (*P* = 0. 01, I^2^ = 40.4%). Third, evident bias also exists accordingly to the publication bias analyses. More case-control studies should be added to conduct the meta-analysis, then to further detect the potential gene-gene and gene-environment association.

In summary, the meta-analyses implied that the *GSTM1* null variant was significantly associated with the susceptibility to colorectal cancer in Asians, which supporting the genetic factors play vital roles in the pathogenesis of colorectal cancer. Further validation studies should be included to solidify the current conclusions.

## Additional Information

**How to cite this article**: Li, J. *et al. GSTM1* polymorphism contribute to colorectal cancer in Asian populations: a prospective meta-analysis. *Sci. Rep.* **5**, 12514; doi: 10.1038/srep12514 (2015).

## Supplementary Material

Supplementary Information

## Figures and Tables

**Table 1:** Detailed information of the eligible studies included in the meta-analysis.

**Reference**	**Population**	**Ethnicity**	**No of cases**	**case/GSTM1**			**control/GSTM1**	
				**null**	**present**	**No of controls**	**null**	**present**
Cong 2014^33^	Chinese men	Asian	264	142	122	317	135	182
Vogtmann 2014^34^	Chinese men	Asian	335	201	134	638	379	259
Hamachi 2013^35^	Japan men	Asian	455	255	200	1052	546	506
Huang X 2012^36^	Chinese	Asian	100	42	58	130	59	71
Koh 2011^37^	Chinese	Asian	480	234	246	1167	526	641
Wang 2011^38^	India	Asian	302	100	202	291	76	215
Nisa 2010^39^	Japan	Asian	685	357	328	778	422	356
Yang 2010^40^	Chinese	Asian	322	189	133	1247	729	518
Yeh 2010^41^	Taiwan	Asian	722	401	321	733	410	323
Piao 2009^42^	Korean	Asian	1829	1004	825	1699	923	776
Lin LM 2008^43^	Chinese	Asian	120	69	51	204	90	114
Huang LR 2007^44^	Chinese	Asian	57	40	17	68	35	33
Xia XP 2007^45^	Chinese	Asian	112	67	45	140	63	77
Yoshida 2007^46^	Japan	Asian	66	36	30	121	62	59
Fan CH 2006^47^	Chinese	Asian	138	80	58	339	188	151
Fu QH 2006^48^	Chinese	Asian	315	229	86	438	321	117
Luo JG 2006^49^	Chinese	Asian	56	20	36	143	48	95
Probst-Hensch 2006^50^	Chinese	Asian	300	132	168	1168	525	643
Chen K 2004^51^	Chinese	Asian	126	69	56	343	188	151
Huang P 2003^52^	Chinese	Asian	82	46	36	82	28	54
Yang J 2003^53^	Chinese	Asian	58	40	18	65	29	35
Seow 2002^54^	Chinese	Asian	213	108	105	1190	537	653
Wu 2002^55^	Taiwan	Asian	356	173	183	278	136	142
Zhu YQ 2002^56^	Chinese	Asian	104	59	45	101	47	54
Saadat 2001^57^	Iran	Asian	46	25	21	131	53	78
Zhang YC 2001^58^	Chinese	Asian	52	22	30	52	25	27
Inoue 2000^59^	Japan	Asian	205	108	97	220	123	97
Zhou JN 2000^60^	Chinese	Asian	55	34	21	62	33	29
Yoshioka 1999^61^	Japan	Asian	106	56	50	100	42	58
Gao JR 1998^62^	Chinese	Asian	19	7	12	70	25	45
Lee 1998^63^	Singapore	Asian	300	128	172	183	89	94
Guo 1996^64^	Chinese	Asian	19	7	12	23	6	17
Katoh 1996^65^	Japan	Asian	103	56	47	126	55	71
