# Supplementary material for: GSTM1 polymorphism contribute to colorectal cancer in Asian populations: a prospective meta-analysis
Source: Sci Rep. 2015 Jul 29;5:12514. doi: 10.1038/srep12514 (PMC4649893; doi:10.1038/srep12514)
Supplement: Supplementary Information [file srep12514-s1.pdf]

Supplementary Materials

GSTM1 polymorphism contribute to colorectal cancer in Asian populations: a prospective meta-analysis

Jing Li[1], Wen Xu[2], Fang Liu[3], Silin Huang[4], Meirong He[5]*
1-5 Guangdong Provincial Key Laboratory of Gastroenterology, Department of Gastroenterology, Nanfang Hospital, Southern Medical University, Guangzhou 510515, Guangdong Province, China

* Corresponding author:
Meirong He: Guangdong Provincial Key Laboratory of Gastroenterology; Tel/Fax: 86-15626451640/020-28905991; E-mail: hemr@263.net

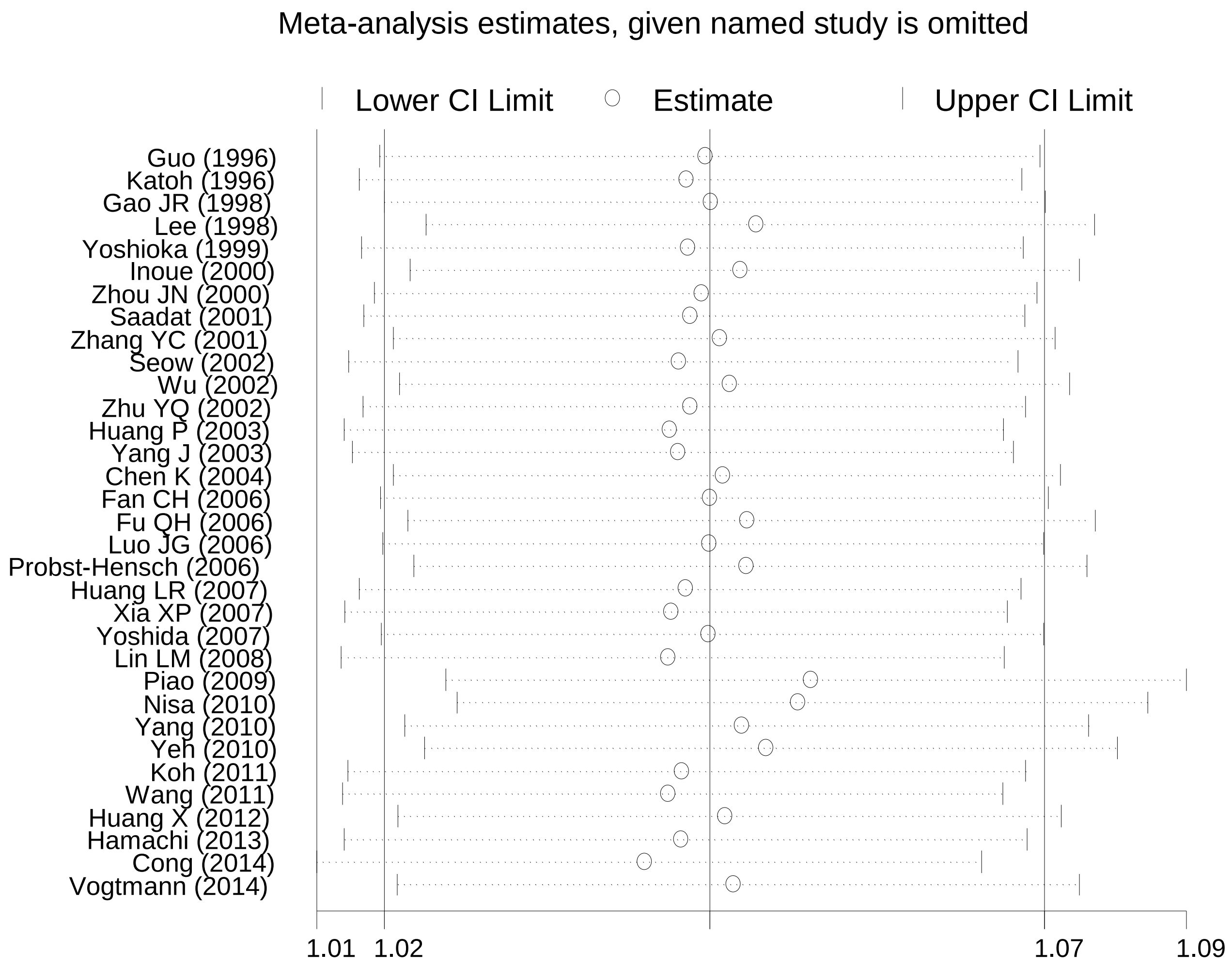

Figure.S1 Result of sensitivity analysis

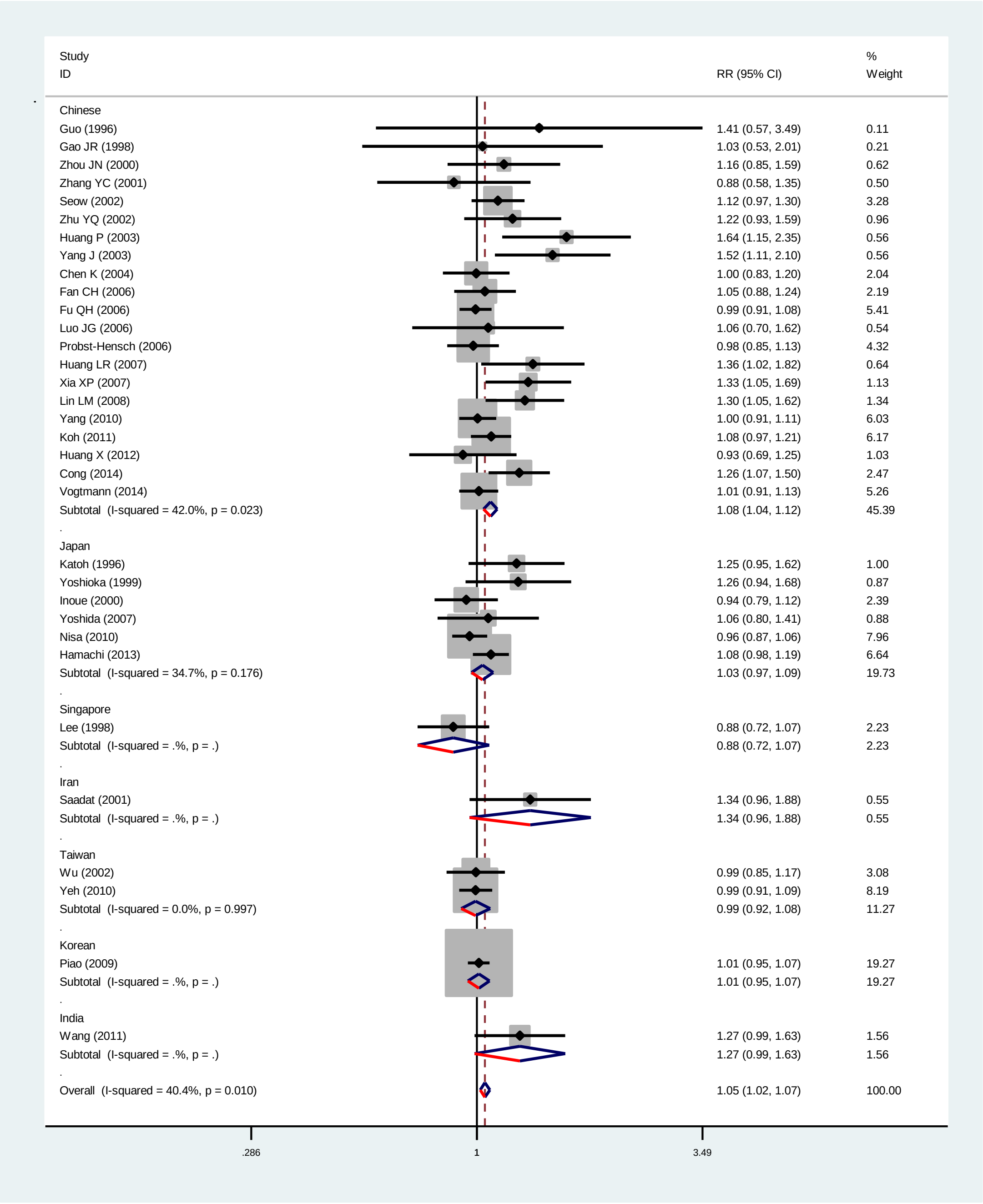

Figure.S2 Sub-group analysis among different countries

```
Begg's Test

  adj. Kendall's Score (P-Q) =     152
          Std. Dev. of Score =   64.54
           Number of Studies =      33
                          z  =    2.36
                    Pr > |z| =   0.019
                          z  =    2.34 (continuity corrected)
                    Pr > |z| =   0.019 (continuity corrected)
```

```
Egger's test
```

Std_Eff	Coef.	Std. Err.	t	P>|t|	[95% Conf.	Interval]
slope	-.0467368	.0289113	-1.62	0.116	-.1057019	.0122283
bias	1.325243	.3773636	3.51	0.001	.5556045	2.094881

Figure.S3 Results of Begg’s funnel plot and the Egger’s regression plot to assess the publication bias

```
Meta-analysis

       |  Pooled      95% CI         Asymptotic      No. of
Method |     Est    Lower   Upper  z_value  p_value   studies
-------+----------------------------------------------------
Fixed  |   0.040   0.014   0.067    3.031    0.002      33
Random |   0.063   0.024   0.102    3.172    0.002

Test for heterogeneity: Q= 53.579 on 32 degrees of freedom (p= 0.010)
Moment-based estimate of between studies variance =  0.004

Trimming estimator: Linear
Meta-analysis type: Fixed-effects model

iteration |  estimate    Tn    # to trim     diff
----------+--------------------------------------
    1     |    0.040    385          6         561
    2     |    0.028    410          8          50
    3     |    0.022    420          9          20
    4     |    0.016    432          9          24
    5     |    0.016    432          9           0

Filled
Meta-analysis

       |  Pooled      95% CI         Asymptotic      No. of
Method |     Est    Lower   Upper  z_value  p_value   studies
-------+----------------------------------------------------
Fixed  |   0.016  -0.009   0.041    1.281    0.200      42
Random |   0.022  -0.022   0.067    0.980    0.327

Test for heterogeneity: Q= 96.241 on 41 degrees of freedom (p= 0.000)
Moment-based estimate of between studies variance =  0.010
```

Figure.S4 Results of Trim and fill test to assess the publication bias
